# Extensive and Persistent Dermal Melanocytosis in a Male Carrier of Mucopolysaccharidosis Type IIIC (Sanfilippo Syndrome): A Case Report

**DOI:** 10.3390/children10121920

**Published:** 2023-12-13

**Authors:** Maurizio Romagnuolo, Chiara Moltrasio, Serena Gasperini, Angelo Valerio Marzano, Stefano Cambiaghi

**Affiliations:** 1 Department of Pathophysiology and Transplantation, Università degli Studi di Milano, 20122 Milan, Italy; 2 Dermatology Unit, Fondazione IRCCS Ca’ Granda Ospedale Maggiore Policlinico, 20122 Milan, Italy; chiara.moltrasio@policlinico.mi.it; 3 Department of Pediatrics, Fondazione IRCCS San Gerardo dei Tintori, 20900 Monza, Italy; 4 Pediatric Dermatology Unit, Department of Clinical Sciences and Community Health, Fondazione IRCCS Ca’ Granda Ospedale Maggiore Policlinico, 20122 Milan, Italy; stefano.cambiaghi@policlinico.mi.it

**Keywords:** congenital dermal melanocytosis, Mongolian spot, itch, hypertrichosis, mucopolysaccharidosis type IIIC, Sanfilippo syndrome, *HGSNAT* mutation

## Abstract

Congenital dermal melanocytosis (DM) represents a common birthmark mainly found in children of Asian and darker skin phototype descent, clinically characterized by an oval blue-grey macule or macules, commonly located on the lumbosacral area. In rare DM cases, when presenting with diffuse macules persisting during the first years of life, it could represent a cutaneous feature of mucopolysaccharidoses (MPS). Extensive congenital DM is actually associated with Hurler syndrome (MPS type I) and Hunter syndrome (MPS type II), although several reports also described this association with MPS type VI and other lysosomal storage disorders (LySD), including GM1 gangliosidosis, mucolipidosis, Sandhoff disease, and Niemann–Pick disease. Here, we present the case of a two-year-old boy presenting with extensive dermal melanocytosis, generalized hypertrichosis, and chronic itch, harboring a heterozygous variant of uncertain significance, NM_152419.3: c.493C>T (p.Pro165Ser), in the exon 4 of *HGSNAT* gene, whose mutations are classically associated with MPS IIIC, also known as Sanfilippo syndrome. This is the first report that highlights the association between extensive congenital DM and MPS type IIIC, as well as a pathogenetic link between heterozygous LySD carrier status and congenital DM. We speculate that some cases of extensive congenital DM could be related to heterozygous LySD carriers, as a manifestation of a mild clinical phenotype.

## 1. Introduction

Congenital dermal melanocytosis (DM) represents a benign birthmark commonly found in children of Asian and darker skin phototype descent. It is characterized by an oval blue-grey macule or macules associated with the presence of melanin-producing dendritic melanocytes in the dermis [1].

Congenital DM is usually located in the lumbar and sacral-gluteal region; the macules usually fade progressively with age and rarely persist up to the age of 6 [1].

It has been hypothesized that the pathogenesis of congenital DM results from the improper migration of melanocytes from the neural crest to the epidermis during fetal development. Melanocytes are embryologically derived from a stem cell population of founder melanoblasts that originate from the neural crest cells (NCC), proliferate in the migration staging area (MSA)-locatedbetween the ectoderm, neural tube, and the somites), and migrate along the dorsolateral pathway, also called the “first wave” of melanocyte migration, to arrive at the basal layer of the epidermis or the hair follicles. Indeed, in this first wave, melanoblasts continue to proliferate, differentiate, and migrate, and only when the melanocytes reach the epidermis is the terminal differentiation complete [2,3]. An alternative source of cutaneous melanocytes, also known as the “second wave” of melanocyte development, arises from nerve-associated Schwann Cell precursors and occurs in the dorso-ventral pathway [3]. Melanocytes are present in the embryo’s dermis by the 10th week of gestation and subsequently migrate to the epidermis between the 11th and 14th weeks [4]. During the transdermal migration, some of the melanocytes can be arrested or undergo an improper macrophage clearance, the latter in terms of apoptosis regulation that orchestrates, together with proliferation, the migration of melanoblasts from precursor melanoblasts, thereby leading to dermal melanocytosis [5,6].

Research data support an association of congenital DM, when presenting with diffuse macules persisting during the first years of life, with lysosomal storage diseases (LySD) [7]. Hurler syndrome, also known as mucopolysaccharidosis (MPS) type I, and GM1 gangliosidosis are the most associated with congenital DM, although several reports also described this association with MPS type VI and other LySD, including mucolipidosis, Sandhoff disease, and Niemann–Pick disease [8].

Here, we present the peculiar case of a two-year-old boy presenting with diffuse dermal melanocytosis, generalized hypertrichosis, and chronic itch, harboring a heterozygous variant of uncertain significance, NM_152419.3: c.493C>T (p.Pro165Ser), in the exon 4 of *HGSNAT* gene, whose mutations are classically associated with MPS IIIC, also known as Sanfilippo syndrome. This is the first report that highlights the association between extensive and persistent congenital DM and MPS type IIIC, as well as a pathogenetic link between heterozygous LySD carrier status and congenital DM.

## 2. Case Presentation

**Figure 1:**
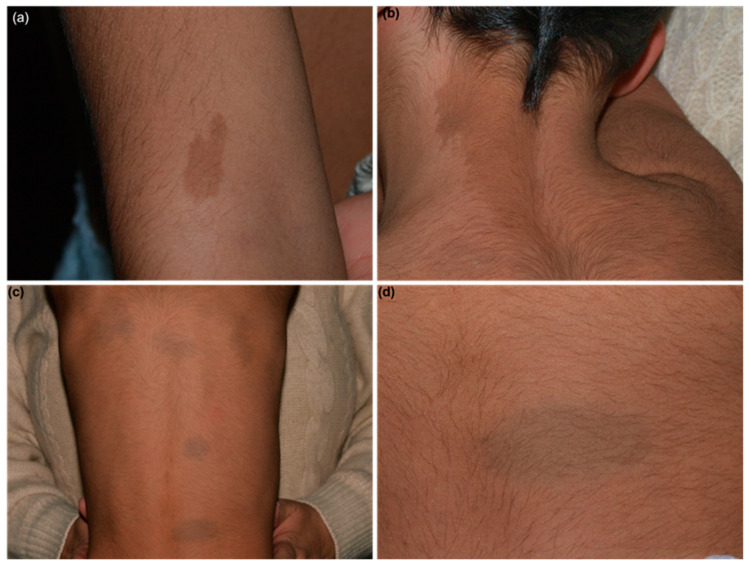
Clinical photographs of the 4-year-old boy showing (**a**) an irregularly shaped light brown patch on the right arm consistent with a jagged cafè-au-lait spot; (**b**) an hyperpigmented (light brown) mosaic along the left latero-cervical region with an irregular lateral border and a sharp edge on the midline; (**c**) diffuse dermal melanocytosis, presenting as multiple blue-grey oval-shaped patches on the back of the patient; and (**d**) a detail of a dermal melanocytosis patch. Generalized hypertrichosis could be appreciated in all photographs.

A two-year-old boy was referred to our pediatric dermatology department for the evaluation of cafè-au-lait spots, which were present since birth. Clinical examination revealed a brown, irregularly shaped patch on the right arm consistent with an isolated benign jagged cafè-au-lait spot and an hyperpigmented patch on the left latero-cervical region with an irregular lateral border and a sharp edge on the midline consistent with a hyperpigmented mosaic (Figure 1a,b). No other cutaneous features suggesting neurofibromatosis (e.g., axillary freckling, multiple cafè-au-lait spots, or soft skin) were detected nor was a familial history of neurofibromatosis reported. The child was in good general health; growth charts, head circumference, and psychomotor development were within the normal ranges for the patient’s age. However, upon clinical examination, diffuse deep-blue oval macules on his back were noted, along with a generalized hypertrichosis (Figure 1c,d). Moreover, his mother stated that the boy suffered from chronic itch, but no cutaneous lesions were found to justify the itch. The pruritus was referred as intermittent and mainly occurred on the hands, back, arms, and legs, occasionally provoking excoriated papules. Clinical examination and the absence of pruritus in the patient’s relatives excluded the diagnosis of scabies.

No personal or family history of atopy and atopic eczema was reported, nor a history of eczematous lesions attributable to irritant contact dermatitis. 

Regional expanded newborn screening (NBS), institutionalized by law between 2016 and 2017 (Law 167/2016; DM 13 October 2016; DPCM 12 January 2017; deliberation XI/110, 14 May 2018), and comprising groups of inherited metabolic diseases including aminoacidemias, organic acidemias, urea cycle defects, fatty acid oxidation disorders, biotinidase deficiency, and galactosemia, was negative. The differential for DM associations including Hurler syndrome, GM1 gangliosidosis 1, mucolipidosis, Niemann–Pick, mannosidosis, phacomatosis pigmentovascularis, and neurological disorders were not tested on NBS, since these conditions are not included in the panel of about 40 diseases provided for in the implementation of law 167/2016. Indeed, to date in Italy, NBS for LySD has been carried out in only two Neonatal Screening Reference Laboratories involved in pilot projects or specific regional regulations. Blood examinations including liver enzymes, creatinine, thyroid function, complete blood count, immunoglobulin E count, anti-transglutaminase antibodies, and direct bilirubin were all normal. A negative perianal scotch tape test ruled out enterobiasis.

Considering the clinical findings (diffuse DM, hypertrichosis, and chronic itch), a cutaneous manifestation of MPS was suspected. No other cutaneous features associated with MPS (e.g., vascular birthmarks, telangiectasias, ivory papules, or plaques) were present.

Moreover, physical examination was unremarkable: there was no history of musculoskeletal and neurological problems, sensorineural hearing loss, macroglossia, facial dysmorphism, dysostosis multiplex, hepatosplenomegaly, gastrointestinal diseases, or inguinal or umbilical hernias. No behavioral, mood, or sleep disorders were detected.

Despite the fact that the urinary glycosaminoglycan (GAG) levels were normal, a Next Generation Sequencing (NGS)-based multi-gene panel for MPS was performed. An EDTA-anticoagulated venous blood sample was collected from the proband. DNA extraction was performed using a QIAamp DNA Mini kit (Qiagen, Valencia, CA, USA) according to the manufacturer’s instructions; subsequently, the DNA concentration was measured using a Qubit 3.0 Fluorometer (Invitrogen, Life Technologies, Van Allen Way, Carlsbad, CA, USA). The captured library was subsequently sequenced on an Illumina^®^ platform and a coverage depth of at least 50× was obtained for all targeted bases. A raw sequence data analysis was performed using designed software (GATK version 4—GATK4). 

The patient showed a heterozygous variant NM_152419.3: c.493C>T (p.Pro165Ser) in the exon 4 of *HGSNAT* (heparan-alpha-glucosaminide N-acetyltransferase) gene, whose mutations are typically associated with MPS IIIC. Based on guidelines from the American College of Medical Genetics and Genomics and the Association for Molecular Pathology (ACMG/AMP), the identified missense variant is predicted to be a variant of uncertain significance (VUS) (PM2_moderate; PP3_supporting), whose frequency in the Genome Aggregation Database (gnomAD) is 0.00018 (0.02%). Algorithms developed to predict the effect of sequence changes on RNA splicing suggest that this variant may disrupt the consensus splice site.

At his last follow-up, two years after the first visit, the boy was in good general health; the birthmarks persisted (Figure 1a–d), and the itch was well-controlled with daily moisturizing and, occasionally, short cycles of mid-potency topical corticosteroids. No sign and/or symptoms of systemic involvement were detected.

## 3. Discussion

Dermal melanocytosis encompasses a group of cutaneous disorders characterized by blue or blue-gray pigmentation of the skin, clinically distinguished from one another by specific location and disease course [8]. 

Congenital dermal melanocytosis, also known with the previous terminology—currently not recommended-“Mongolian spot”, is one of the most frequent neonatal pigmented lesions manifest with blue-grey macule(s), with a round or oval shape and commonly located in the lumbar and sacral-gluteal region [1]. Congenital DM presents the highest prevalence in Asian and Black populations [9] and typically occurs at birth or shortly thereafter, with most cases having the lesions disappear by the age of 1, rarely persisting after the age of 6 [2]. Although the diagnosis is strictly clinical, histological examination can be a valid tool to confirm and support the diagnosis; spindle-shaped melanocytes located between collagen fibers deep within the dermis are typically observed [1].

A differential diagnosis of blue and/or blue-gray lesions includes other DMs such as Nevus of Ota, Nevus of Ito, and also congenital and acquired blue nevus [10,11].

All dermal melanocytoses share a common pathogenetic scenario, in which the improper migration and/or inappropriate clearance (by macrophages) of melanocytes occurs during fetal development [6]. During transdermal migration (from the neural crest to the developing epidermis), some of the melanocytes may be arrested in the dermis, thereby leading to dermal melanocytosis [12]. 

As mentioned above, congenital DM usually fades progressively with age and rarely persists up to the age of 6 [1]. Two main hypotheses have been formulated to explain this clinical course: the theory of regression and the local hindrance phenomenon [4]. Dermal melanocytes are wrapped in a protective extracellular fibrous sheath that is gradually lost, starting from fetal life onwards and peaking in early childhood; since these cells are scattered among the collagen bundles, body growth can act as a driving force for the disruption of the filamentous sheath, possibly leading to melanosome phagocytosis by dermal melanophages [13,14]. In contrast, a local abundance of melanocyte-stimulating growth factors, a defective regulation of melanocyte proliferation, and genetic factors are thought to be responsible for the persistence of dermal melanocytes. 

Research data support an association of congenital DM (when presenting with diffuse macules persisting during the first years of life) and inborn errors of metabolism [15,16,17]. It has been demonstrated that lysosomal storage diseases (LySDs), particularly Hurler syndrome, also known as mucopolysaccharidosis (MPS) type I, and GM1 gangliosidosis, can be associated with extensive and persistent congenital DM [3,18,19]. In comparison to other LySDs, these two conditions are more commonly associated with a severe neurological impairment of earlier onset in infancy. It can be assumed that abnormally accumulated dermal metabolites bind tyrosine kinase type receptors, which in turn activate the Nerve Growth Factor (NGF) signaling pathway, which is responsible for melanocytes chemotaxis, finally interfering with the normal melanocyte migration towards the epidermis [8].

Several reports also described this association with other LySDs, including mucolipidosis, Sandhoff disease, Niemann–Pick disease, Hunter syndrome (MPS type II), and MPS type VI [3]. 

MPS represents an heterogenous group of inherited—mostly autosomal recessive—LySDs, characterized by the absence or deficiency of enzymes involved in the glycosaminoglycan (GAG) catabolism cascade, resulting in the subsequent accumulation of GAGs in cells and body organs [20]. Depending on the affected gene, seven types of MPS with thirteen subtypes are reported, thus encompassing a wide clinical and molecular heterogeneity. Common clinical features are represented by facial dysmorphism, intellectual disability, and skeletal, gastrointestinal, cardiovascular, and respiratory involvement. Cutaneous features are not disease-specific, and may include extensive DM, hypertrichosis, or generalized hirsutism, extensive telangiectasias, acrosclerosis-like skin thickening, particularly on the dorsa of the hands, and white to ivory papules, which could be coalescing and forming plaques and occasionally nodules symmetrically distributed on the upper back [7]. 

As mentioned above, extensive and progressive congenital DM is classically associated with Hurler syndrome (MPS type I) and GM1 gangliosidosis [7]: in a large cohort of 50 Indian families affected by GM1 gangliosidosis, an autosomal recessive disease caused by a deficiency of lysosomal enzyme β-D-galactosidase, extensive DM represented a cutaneous feature in 65% of the patients included. Interestingly, the authors observed that the number of hyperpigmented macules increased with the progression of the disease, although no genotype–phenotype correlation or association with the overall disease severity was evident [21]. Similar results were seen in other Indian and Chinese cohorts [22,23], thus supporting the association of extensive congenital DM with GM1 gangliosidosis in Asian and darker skin phototypes patients from India.

In addition, a retrospective study on a cohort of Japanese patients estimated that 78% of children with Hunter syndrome (MPS type II) also presented an extensive congenital DM, which persisted into adulthood with a faint grey color as opposed to the deep blue pigmentation of the younger patients [18]. 

Various reports also described the association of DM with other MPS subtypes and LySDs, including Sandhoff disease and Niemann–Pick disease [24,25]. Ashrafi and colleagues [24] described a 18-month-old Iranian girl affected by Sandhoff disease, caused by the inherited deficiency of the enzyme hexosaminidase B, who presented an extensive DM located on the trunk, abdomen, sacro-coccygeal region, arms, and legs associated with severe neurological impairment. In the same work, the authors also reported a 4-year-old girl with a diagnosis of MPS VI, presenting with diffuse DM on the back, shoulder, and buttocks, typical coarse facies, brachydactyly, and hepatosplenomegaly [24]. Su and colleagues [25] reported the occurrence of extensive and spotted DM on the back, shoulder, and arms in a 14-month-old Chinese girl affected by mucolipidosis type II, an inherited LySD due to uridine diphospho-N-acetylglucosamine enzyme deficiency, with similar skeletal radiological and clinical findings to Hurler’s disease but no evidence of mucopolyisacchariduria [25]. 

Our patient presented an extensive deep-blue congenital DM on the back and shoulders, along with generalized hypertrichosis and a chronic, sine materia itch, occasionally resulting in excoriated papules on the back and dorsum of the hands. All these cutaneous features have been associated with MPS [7].

To the best of our current knowledge, no cases of extensive congenital DM and MPS type IIIC have been previously reported, as no association has been found between heterozygous LySD carrier status and DM. Regarding the latter issue, we can postulate that some cases of extensive DM could be associated with heterozygous LySD carriers, as a manifestation of a mild clinical phenotype.

Further clinical reports on larger cohorts along with molecular studies, in terms of functional validation on model organisms and/or in vitro, are necessary to confirm our observation. If validated, this association could help clinicians in LySD carrier screening and counseling.

## 4. Conclusions

Although congenital dermal melanocytosis represents a common and innocent birthmark, when it appears more widely distributed, also affecting the extremities and trunk in addition to the dorsal and lumbar area, persisting and/or increasing after the first years of life, an associated inborn error of metabolism should be suspected. In addition to extensive DM, other cutaneous features such as hypertrichosis, white to ivory papules coalescing into plaques giving the appearance of “pebbly skin”, and a chronic itch which could result in skin thickening (particularly on the acral sites), should also raise the suspicion of an LySD, in particular an MPS [7]. Moreover, if further validated, this assumption could also apply to carrier subjects, thus enhancing screening and counselling for this heterogenous group of rare diseases.

## Data Availability

The data are contained within the article.
